# De novo complex intra chromosomal rearrangement after ICSI: characterisation by BACs micro array-CGH

**DOI:** 10.1186/1755-8166-1-27

**Published:** 2008-12-23

**Authors:** Serdar Kasakyan, Laurence Lohmann, Azeddine Aboura, Mazin Quimsiyeh, Yves Menezo, Gerard Tachdjian, Moncef Benkhalifa

**Affiliations:** 1 ATL R&D laboratory & Eylau Laboratory, UNILABS Group, Paris, France; 2 Laboratoire Clement, Le Blanc mesnil, France; 3 Service de Biologie et Génétique de la Reproduction, INSERM U935, Hôpital A. Béclère, Clamart, France; 4 SiParadigm Laboratories, 690 Kinderkamack Rd, Oradell, NJ, USA

## Abstract

**Background:**

In routine Assisted Reproductive Technology (ART) men with severe oligozoospermia or azoospermia should be informed about the risk of de novo congenital or chromosomal abnormalities in ICSI program. Also the benefits of preimplantation or prenatal genetic diagnosis practice need to be explained to the couple.

**Methods:**

From a routine ICSI attempt, using ejaculated sperm from male with severe oligozoospermia and having normal karyotype, a 30 years old pregnant woman was referred to prenatal diagnosis in the 17^th ^week for bichorionic biamniotic twin gestation. Amniocentesis was performed because of the detection of an increased foetal nuchal translucency for one of the fetus by the sonographic examination during the 12^th ^week of gestation (WG). Chromosome and DNA studies of the fetus were realized on cultured amniocytes

**Results:**

Conventional, molecular cytogenetic and microarray CGH experiments allowed us to conclude that the fetus had a *de novo *pericentromeric inversion associated with a duplication of the 9p22.1-p24 chromosomal region, 46,XY,invdup(9)(p22.1p24) [arrCGH 9p22.1p24 (RP11-130C19 → RP11-87O1)x3]. As containing the critical 9p22 region, our case is in coincidence with the general phenotype features of the partial trisomy 9p syndrome with major growth retardation, microcephaly and microretrognathia.

**Conclusion:**

This de novo complex chromosome rearrangement illustrates the possible risk of chromosome or gene defects in ICSI program and the contribution of array-CGH for mapping rapidly de novo chromosomal imbalance.

## Background

In Assisted reproductive technology (ART), male with severe oligozoospermia or azoospermia should be offered genetic/clinical counselling for informed consent about the risk of de novo congenital or chromosomal abnormalities before ICSI [1]. The precise risks of genes imprinting and childhood cancer from ART is still unclear but can not be ignored [2]. Furthermore, gene expression and methylation status in animal embryos can be affected by changing the culture conditions during ART processes [3].

Partial trisomy 9p is a frequently described chromosome abnormality. The partial trisomic fragments of the published cases are heterogeneous causing unusual presentations of characterized phenotypes [4-6]. The cases with such abnormalities usually present considerable diagnostic difficulties both clinically and cytogenetically.

In clinical cytogenetics, the precise identification of the chromosomal abnormality is a key factor when considering genotype-phenotype correlation. Advances in molecular cytogenetics have allowed more precise analysis of complex chromosomal rearrangements, especially with FISH techniques, spectral karyotyping and conventional CGH. The developments of the array-CGH the accuracy of identification of complex chromosomal anomalies, such as unbalanced intrachromosomal rearrangements [7,8].

Microarray CGH technology was recently applied to constitutional chromosomal abnormalities demonstrating its sensitivity parallelly with the use of other techniques to detect submicroscopic chromosomal aberrations [9-13]. This technology can therefore be applied in prenatal diagnosis to reveal, with a higher resolution, chromosomal imbalances in malformed foetuses [14]. For molecular karyotyping Array CGH (A-CGH) methods are superior to FISH in not requiring suitable nuclear preparations and in not being limited to probes used. They are also superior to routine metaphase CGH because of their higher resolution, easier interpretation and hold the promise and routine diagnostic tool to identify visible and submicroscopic chromosome abnormalities [12,15,16].

From a routine ICSI attempt, using ejaculated sperm from male with severe oligozoospermia and having normal karyotype, we report the first prenatal diagnosis of a *de novo *pericentric inversion and duplication of a large segment of the short arm of a chromosome 9, characterized by CGH array and FISH. Risks of de novo chromosomal and genetics disorders after ART are discussed.

## Methods

### Case report

**Figure 1:**
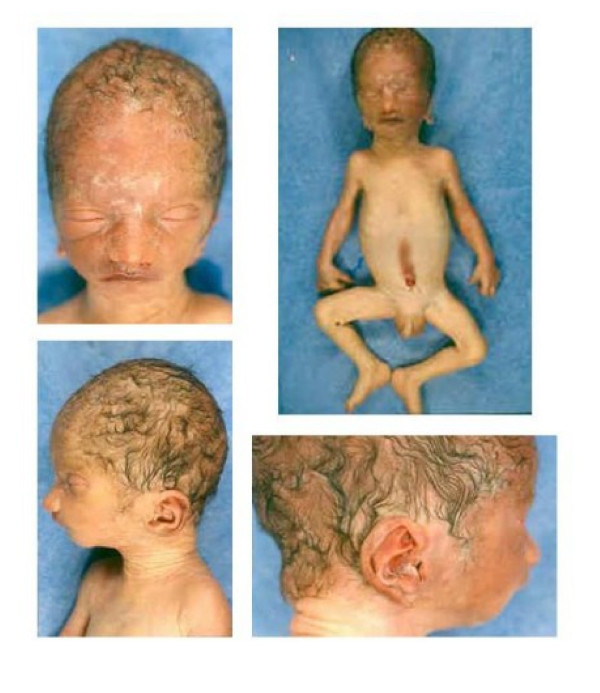
**Phenotype of the fetus**. A) Face with hypertelorism, B) general view of the fetus showing microcephaly and contracted hands, C) profile showing microretrognathia and short upper philtrum, D) low-set asymetric malformed protruding ears.

Intra Cytoplasmic Sperm Injection was applied for oligoazoospermia indication. Three embryos were transferred at day 3. The transfer led to twin pregnancies. Amniocentesis was performed because of the detection of an increased foetal nuchal translucency for one of the fetus by the sonographic examination during the 12^th ^week of gestation (WG). The parents were both healthy, non-consanguineous. There was no family history of genetic or congenital disorders. The couple had a spontaneous abortion at 24^th ^WG, without any diagnosis found (normal autopsy and normal foetal karyotype). Ultrasound examination at 21^th ^WG revealed for this fetus, brachycephaly, short femur, intra uterine growth retardation (<3^rd ^percentile), little low set ears, little stomach and increased amniotic fluid volume. The foetal karyotype showed a *de novo *abnormal chromosome 9. The parents were informed about the poor prognosis of this fetus. After interdisciplinary discussion, selective termination of pregnancy was performed at 34^th ^WG according to French law. The normal twin was eutrophic with a normal clinical examination. The abnormal fetus was referred to the department of foetal pathology. Anatomic examination showed microcephaly and dysmorphic features such as low set, malformed protruding asymmetric ears, short philtrum, and microretrognathia, contracted hands with hypoplasia of the medium phalange of the fifth fingers, left foot in varus, hypertelorism, mild bilateral urethral dilatation and major growth retardation (fig. 1).

### Cytogenetic investigations

Amniocytes were cultured using standard *in situ *technique. Peripheral blood samples of both of the parents and of the fetus were stimulated with PHA and cultured 72 hours before harvesting. Foetal skin sample was treated and cultured using standard protocols. Chromosome studies of the fetus were realized on cultured amniocytes and cultured skin samples using G-banding, R-banding and C-banding techniques performed according to the standard procedure. The chromosomes from peripheral blood samples of the parents were analysed from cultured lymphocytes.

### Array CGH – DNA microarray analysis

We used human genomic micro arrays containing 2600 BAC/PAC clones with an average of 1 MB resolution along the human genome (Human BAC Array-1 MB system, Spectral Genomics Inc., Houston, Texas, USA). This micro array includes subtelomeric regions as well as critical areas spaced roughly 1 Mb along each of the human autosomes as well as the X and Y chromosomes. Data on the DNA clones are available in the public records including their map positions as identified by FISH and/or other techniques .

Total genomic DNA was obtained from cultured amniocytes by a standard extraction using phenol/chloroform. Test and control DNA samples (2 μg each) were digested overnight with 80 units of EcoRI at 37°C and then purified by Zymo Research's column (Orange, CA, USA). The test and reference DNAs were labelled with Cy3 and Cy5 using random prime labelling kit (Invitrogen, France) to obtain a major labelled probe size between 100 to 500 bp.

For the hybridisation solution, Cy5 labelled Test DNA and Cy3 labelled reference DNA samples were mixed with 65 ug of Cot-1 DNA and 35 μg of shared salmon testes DNA then the mix was precipitated and washed with ethanol. The same experiment was repeated with Cy3 labelled test and Cy5 labelled reference test. This forward and reverse hybridization switching of dyes helps address issues related to dye specificity and strength. The pellets were dissolved homogeneously in 10 μl of distilled water and mixed with 50 μl of hybridisation solution (50% formamide, 10% dextran sulphate in 2 × SSC). The hybridisation mix was denatured at 73° for 12 min and followed by 40 min at 37°C for annealing.

The forward and reverse hybridization reactions were added on duplicate micro array slides and placed at 37°C for 16 hrs. After hybridization, slides were washed briefly at room temperature in 2 × SSC/0.5% SDS to remove the hybridization solution, then 20 min in 2 × SSC/50% deionised formamide at 50°C with shaking. The washing steps were repeated at 50°C with shaking in 0.2 × SSC/0.1% NP40 for 20 min and 0.2 × SSC for 20 min. Finally the slides were rinsed briefly with distilled water at room temperature and centrifuged for 3 min at 500 g for complete drying.

Hybridized micro arrays were analysed with GenePix 4000B scanner (Axon Instruments Inc., Union City, CA, USA). Cy3 and Cy5 images were scanned separately through two different channels. Two 16 bit TIFF images were created per array. Then the obtained data were analysed by Spectralware 1.0 software (Spectral Genomics Inc., Houston, Texas, USA). The software recognizes the regions of fluorescent signal determine signal intensity and compile the data into a spreadsheet that link the fluorescent signal of every clone on the array to the clone name, its duplicate position on the array and its position in the genome. The software was also used to normalise the Cy5:Cy3 intensity ratios for each slide and each data point. Slide was normalised such that the summed Cy5 signal equal the summed Cy3 signal. The normalised Cy3:Cy5 intensity ratios were computed for each two slides and plotted together for each chromosome. The linear order of the clones is reconstituted in the ratio plots consistent with an ideogram, such that the p terminus is to the left and the q terminus is to the right of the plot.

Chromosomal areas are interpreted as overrepresented when the ratio exceeds 1.2 which shows DNA copy number gains or 1.5 which shows DNA copy number amplifications with the blue ratio plot showing a positive deviation (upward) and red ratio plot showing a negative deviation (downward), reversely 0.8 and 0.5 for DNA copy number losses or deletions respectively with the blue ratio plot showing a negative deviation and red ratio plot showing a positive deviation. On the ratio plots only the deviations representing a mirror effect considered as significant due to reverse and forward hybridization switching assays. As CGH recognizes only proportional changes in copy number, the ratio profiles do not indicate the absolute copy number changes. A ratio of 1.5 indicates at least a 100% increase in the copy number of an entire chromosome arm or of a region of a chromosome the size of a chromosome band (e.g. chromosomal trisomy). When a DNA copy number increase is restricted to a small chromosome area representing, for example, amplification of a single gene, then the copy number increase has to be at least 1 Mb which is the resolution of the micro array used.

### Fluorescence in Situ Hybridisation (FISH)

FISH analyses were performed on metaphase spreads from both cultured fetal skin and fetal blood with, whole chromosome painting probe specific for chromosome 9 (Vysis, Rungis, France), partial chromosome painting (PCP) probes specific for 9p and 9q arms (Cytocell, Compiegne, France) and BAC/PAC clones specific for the 9p and 9q chromosomal regions. The satellite I (Cytocell) and satellite III probes (Appligen Oncor, Illkirch, France) specific for chromosome 9 were also used. The BAC DNAs were labelled by nick-translation using a FITC-dUTP nucleotide or a Rhodamine-dUTP nucleotide (Roche Diagnostics, Meylan, France). Results were analyzed using a Zeiss Axioplane microscope connected to a Photometrics CCD camera and evaluated with the aid of IPLab-Spectrum 3.0 software (Carl Zeiss S.A.S., France).

## Results

### Conventional cytogenetic analysis

**Figure 2:**
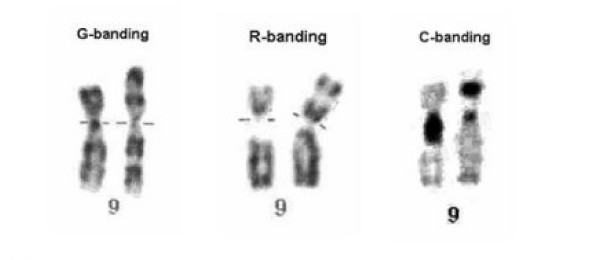
**Conventional cytogenetic analysis results of the fetus**. G and R-banding results showing the addition of chromosomal materiel in the short arm of one chromosome 9 (der(9)). C-banding results showing an presence of heterochromatin into the der(9).

Cytogenetic analysis of amniocytes revealed a 46,XY,9p+ karyotype for the abnormal fetus. R- and G-banding results showed the presence of excedent material in the short arm of one chromosome 9. C-banding showed the presence of heterochromatin into the short arm of the derivative chromosome 9 (fig. 2). Analysis of peripheral blood lymphocytes of the parents showed normal karyotypes.

### Array CGH – DNA microarray analysis

**Figure 3:**
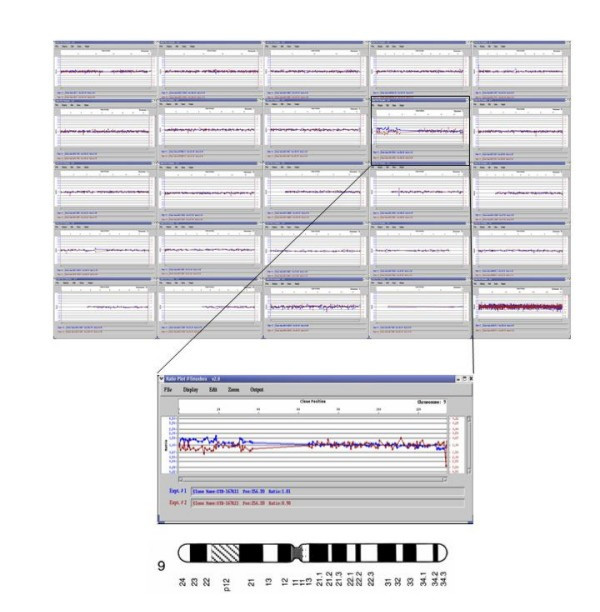
**Profile of chromosome 9 using a human 1 Mb genomic micro array showing a gain of the 9p24-p22.1 region**.

The array-CGH profile for chromosome 9 showed a gain between the chromosomal bands 9p24 and 9p22.1 (fig. 3). There were no copy number changes on the rest of the genome of the fetus.

### FISH analysis

**Figure 4:**
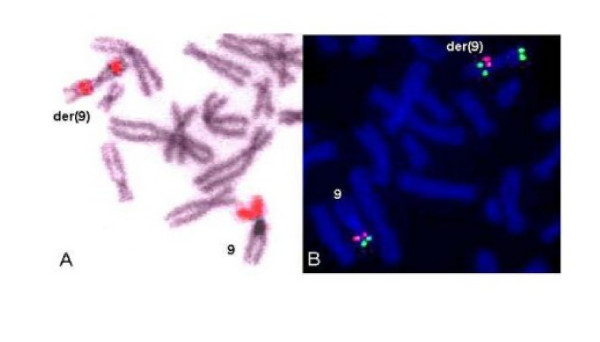
**FISH results showing an inversion and duplication of the chromosomal region 9p24 and 9p22.1**. A) FISH with painting probe specific for 9p arm (red). B) FISH with RP11-39K24 BAC clone (green) corresponding to 9p24.1 and RP11-149I2 BAC clone (red) corresponding to 9p21.3.

FISH results with a whole-chromosome 9 painting probe had shown rearranged chromosome 9 being entirely hybridised and that there were no other chromosomes implied in the chromosomal rearrangement. The PCP (partial chromosome painting) probe specific for the short arm of the chromosome 9 showed two hybridization areas on the short arm and on the long arm of the derivative chromosome 9 (fig. 4A). The PCP probe specific for the long arm of the chromosome 9 showed two hybridization areas on the short arm and on the long arm of the derivative chromosome 9.

Specific probe for the centromere of the chromosome 9 (satellite I) showed normal hybridization signals on both normal and abnormal chromosomes 9. The probe specific for the heterochromatin of the chromosome 9q (satellite III) showed a splitted hybridization signal on the abnormal chromosome 9. FISH results showed an inverted duplication of the 9p22.1-9p24 chromosomal region (figure 4B). The results of the FISH experiments are presented in table 1.

**Table 1:** FISH results showing the presence of inversion and duplication of 9p22.1-9p24 region on the derivative chromosome 9.

			FISH	
FISH probes	locus	array-CGH	normal 9	der(9)

PCP 9p	9p	Gain 9p22.1-9p24	+	++ (inv/split)

PCP 9q	9q	normal	+	++ (inv/split)

Satellite I	Centromere 9	non analyzed	+	+

Satellite III	9q heterochromatine	non analyzed	+	++ (inv/split)

9p telomere	9p24.3	normal	+	+

9q telomere	9q ter	normal	+	+

RP11-149I2	9p21.3	non analyzed	+	+ (inv)

RP11-109M15	9p22	gain	+	++ (inv)

RP11-91E3	9p23	gain	+	++ (inv)

RP11-39K24	9p24.1	non analyzed	+	++ (inv)

Normal: +

Duplication: ++

Inversion: inv

Split: splitted FISH signal

In summary, conventional and molecular cytogenetic experiments allowed us to conclude that the fetus had a *de novo *pericentromeric inversion associated with a duplication of the 9p22.1-p24 chromosomal region, 46,XY,invdup(9)(p22.1p24) [arrCGH 9p22.1p24 (RP11-130C19 → RP11-87O1)x3].

## Discussion

Men with severe oligozoospermia or azoospermia should be offered genetic/clinical counselling for informed consent about the risk of de novo congenital or chromosomal abnormalities and somatic karyotyping before ICSI [1]. Jozwiak et al [17] showed that ICSI babies carry a significant increase risk of an abnormal karyotype compared to the children's conceived by natural pregnancies. However this increase risk is similar among the different infertility group. In early abortion before 12 weeks of pregnancies conceived by IVF, the de novo chromosome aneuploidy is a major factor affecting normal embryonic development [18]. The precise risks of genes imprinting and childhood cancer from ART is still unclear but can not be ignored [2].

From 23 women who had conceived by IVF and had abortion before 12 weeks, Philipp et al [18] reported 17 of 23 specimens (73.9%) with chromosome abnormalities using classical cytogenetics chorionic villi investigation. The majority of observed chromosome abnormalities were numerical such as monosomy and trisomies (including 1 trisomy 9) with only one case of structural aberration leading to a trisomy.

Most of the genetic risk in ICSI is linked to the higher frequency of chromosomal abnormalities in men with severe oligoasthenoteratozoospermia or azoospermia, genetic screening and counselling should be given before ICSI [19]. Most studies of children conceived in vitro have shown a negligible or only a slight excess risk of major and minor birth defects [20,21]. However, the possibility of those chromosomal abnormalities after ICSI may also be due to disorders of mainly epigenetic origin since the imprinting of genes plays an important role during preimplantation embryo development [22]. Furthermore, gene expression and methylation status in animal embryos can be affected by changing the culture conditions during ART processes [3]. Nevertheless, there are no any imprinted genes known on chromosome 9p according to the Imprinted Gene Catalogue . In the literature, no any data presented about *de novo *9p chromosomal arm rearrangements in ICSI conceptions, which is the case in our study. In the literature there are a few data concerning prenatal chromosome analysis after ICSI. Bonduelle *et al. *[20] report that different structural anomalies are found in 8 cases from 1437 (0.5%) abnormal fetal karyotypes in prenatally tested ICSI fetuses [20]. The cytogenetic analysis of 475 fetus conceived by ICSI showed 4.42% of chromosome abnormalities including 1.83% of structural aberrations [23]. This rate of de novo abnormalities is nearly 2 fold higher than the percentage reported by Bonduelle et al [20]. At present, we cannot draw any significant conclusions from present data about the influence of ICSI in producing chromosomal abnormalities. Moreover, a germinal mosaic in the father cannot be excluded in this case but unfortunately sperm analysis was not available to perform this research. This uncertainty surely justifies the need for studying human embryos and the survey of this work requiring a large international effort. This research must be continued to properly address the issue of safety of ICSI.

Partial trisomy 9p has been reported in few cases and more frequently as a result of different types of translocations either de novo or inherited [24-29]. The prognosis of partial trisomy 9p remains very pejorative and the termination of pregnancy is the most often proposed solution [24]. Partial trisomy 9p is characterized by microcephaly with large anterior fontanel and micrognathia, malformed protruding ears, small sunken eyes, acentric displacement of pupils, hypertelorism, enophthalmos, downslanting palpebral fissures, large bulbous nose, short philtrum, down-curved corners of the mouth, short or webbed neck, small hands, clinodactyly, hypoplasia of the phalanges, short and triangular distal phalanges of the thumbs, spinal lordosis and scoliosis, delayed skeletal maturation, congenital heart defects, kidney abnormalities, mental retardation and frequent perinatal mortality. Our case is in coincidence with the general phenotype features of the trisomy partial 9p syndrome with major growth retardation, microcephaly, microretrognathia, little low set and malformed protruding ears, short philtrum, hypertelorism and hypoplasia of the phalanges. Up to now, prenatal diagnosis of partial trisomy 9p has been described in 6 cases [24,30-33]. Ultrasound signs reported in these cases were intrauterine growth retardation (2/6), cerebral anomalies (4/6), brachycephaly (1/6). Nevertheless, this phenotypic variability can be explained by the fact that the partial trisomy 9p was also associated with another chromosomal anomaly in 5 of these 6 published cases. Moreover, the sizes of partial trisomic 9p fragments in these prenatal cases are also heterogeneous participating to the phenotypic variability [4]. Previously reports suggest that 9p22 may be responsible for the observed phenotype in 9p duplication cases [34-36] have suggested that the 9p duplication critical region lies within a 6-Mb portion of 9p22 [36]. In our case, the duplicated chromosomal region includes the 9p22 band. To our knowledge, our case is the first report of a fetus with a partial trisomy 9p associated with a pericentric inversion.

Advances in molecular cytogenetics have allowed more precise analysis of complex chromosomal rearrangements, especially with FISH techniques, spectral karyotyping and conventional CGH. The publication of the draft sequence of human genome and recent development of the array-CGH dramatically increased the accuracy of identification of complex chromosomal anomalies, such as intrachromosomal rearrangements.

Array comparative genome hybridization (Array CGH) using spotted bacterial artificial chromosomes (BACs), phage artificial chromosomes (PACs), cDNAs or oligonucleotides was developed to detect chromosomal copy number changes on a genome-wide and/or high resolution scale [37,38]. Array CGH has the potential to be applied in clinical diagnostics and may address many of the limitations of both conventional cytogenetics, FISH or PCR [39]. CGH array limitations in a clinical cytogenetics include its inability to detect polyploidy or balanced chromosome abnormalities. Polyploidy can be easily detected by FISH, micro satellite analysis, or flow cytometry. Balanced translocations will still be detected using classic cytogenetics or FISH.

Microarray based CGH has proven to be specific, sensitive, and rapid for whole genome analysis in a single experiment [40,41] and without cell culture [13]. Microarrays also provide distinct advantages over conventional and molecular cytogenetics (pre and post natal, cancer and oncology) analysis because they have the potential to detect the majority of microscopic and sub microscopic chromosomes changes from any DNA sources with or without whole genome amplification [42,43].

Other advantage of array-CGH is the increase in resolution that can be achieved compared to chromosome-based CGH. For BAC arrays, the limit of resolution is on the order of 100–200 kb with full genome coverage using a minimal tiling path of overlapping clones. Constitutional deletions as small as 40 kb have been detected using an array encompassing a 7 Mb interval of chromosome 22 with 90% coverage [44]. A-CGH can also provide a technically less demanding and more sensitive assay than classic CGH or even routine cytogenetics. This is because it is more amenable to automation and provides finer resolution and better quality controls. Thus, it appears likely that in the next few years, array based CGH will become routinely used in clinical cytogenetics

## Conclusion

Our study confirms the importance of the combination of different techniques like chromosome banding, FISH and array-CGH to completely analyse the complex chromosomal rearrangements. FISH was essential in the confirmation of the cytogenetic abnormality and further delineation of the chromosomal disorder. Array-based CGH has much greater multiplexing capabilities than FISH but our study shows the irreplaceable role of FISH technology in molecular cytogenetic diagnostics. This de novo complex chromosome rearrangement illustrates the possible risk of chromosome or gene defects in ICSI program and the contribution of array-CGH for mapping rapidly de novo chromosomal imbalance.

## Competing interests

The authors declare that they have no competing interests.

## Authors' contributions

All the authors contributed to and have approved the final version of the manuscript
